# Beyond Coronavirus: the metamorphosis as the essence of the phenomenon

**DOI:** 10.1007/s11019-021-10063-y

**Published:** 2022-01-26

**Authors:** Filomena Pietrantonio

**Affiliations:** Internal Medicine Department, Ospedale dei Castelli, Azienda Sanitaria Locale Roma 6, Ariccia, RM Italy

**Keywords:** Coronavirus, Metamorphosis, Health policy, Public health, Emergency preparedness

## Abstract

This paper is an insight on a front-line doctor’s experience of Coronavirus in Italy, in an Internal Medicine ward transformed to a COVID-19 ward. Using content analysis were analyzed 52 destructurated interviews to “Covid clinicians” in the “Ospedale dei Castelli” hospital structure in Rome, Italy. Thematic analysis was performed to recognize common topics in the interviews. Finally, a correlation between the 5 Ovid’s forces (love and thirst for knowledge—positive ones—anger, envy and fear—negative ones) and Narrative Medicine scenarios (Physician and Patients, Physician and Society, Physician and Self, Physician and Colleagues) is described. Coronavirus is a “tsunami” by confrontation with the poet Ovid’s five driving forces. Covid-19 never gave health-workers a chance to draw breath for a moment themselves, as presentation and treatment hypotheses changed at dizzying speed, constantly forcing them to modify and adapt established procedures and behavior as the pandemic evolved. Every scenarios present a correlation with at least one positive and one negative force (Physician and Patients: love and anger, Physician and Self: Fear and Thirst for Knowledge, Physician and Colleagues: Thirst for Knowledge and Envy, Physician and Society: Love, Fear and Anger). Many healthcare workers who came face-to-face with the magnitude of this emergency are able in some way to contextualize the social implications of this experience, which paradoxically has some positive aspects, having let them discover newfound courage, resourcefulness, and hope. Negative forces result from too strong positive emotions and they are a signal of deteriorated relationships and an alarm bell of clinician’s burn-out. Covid era has been defined by the lack of emergency preparedness, together with lack of international coordination and media reports generating terror. People can defeat Covid by combatting against terror of the heart, by finding passion and courage and by dealing honestly with the fear of disease and death.

## Introduction

The following reflections originate from a meditation on the meaning of the Literature today and whether within it lie the tools to effectively interpret this emergent and unexpected healthcare phenomenon not only using intellect but also in terms of our inner perception or “interior duration”, which runs parallel to physical time and events. This hidden dimension must not be forgotten given the unpredictable, ephemeral, and unstable nature of this healthcare emergency. Virtual communication, mass media and the effects of globalization tend to minimize or distort the dimensions of our inner space; by “walking in the vineyard of the text” (Illich 1994) or dedicating time not only to the sound bites but also to the subtleties of this unfamiliar clinical and personal situation, we can reconquer this essential part of ourselves to return replenished to the fray.

Ovid’s Metamorphosis. “I want to speak about bodies changed into new forms. You, gods, since you are the ones who alter these, and all other things, inspire my attempt, and spin out a continuous thread of words, from the world's first origins to my own time” (Publio Ovidio Nasone. Metamorphoses in Italian verses by M.P. Castagnoli 1853).

Since the beginning of the most shocking pandemia of our time (Doshi 2020), Ovid’s verses have been fixed in mind. In times of upheaval and chaos, the underlying common denominator was always “the wrath of the gods”, who wished to demonstrate to man how the power of creation greatly exceeds man’s capacity to transform their reality.

Each episode recited by the Poet stems from one of the five driving forces recognized by the ancient world: Love, Anger, Envy, Fear and Thirst for Knowledge.

These five forces as described by Ovid have been our compass in navigating Covid pandemic phenomenology, using Narrative Based Medicine (NBM) for analyzing the four major narrative scenarios: physician and patients, physician and self, physician and colleagues, physician and society. The great impact of pandemic worldwide can actually be read in terms of the distortions evoked in these four fundamental relation schemes.

Narrative based medicine is the passage by the “cure” to the “care”, an interaction health practitioner-patients who treats the person who’s suffering from an illness (the patients, by Greek “πάσχω” [paːs.kʰɔː], to suffer) by listening closely to his story and doesn’t simply look at diseases. The Author use NBM to reconnects with her patient, manifesting interest on how the patient’s life is affected by illness, not only on how it can be effectively treated. NBM is presented to counteract the pitfalls of evidence-based medicine (EBM). Narrative Based Medicine can foster a better care while taking into account the patient’s story on the way illness is affecting the quality of his everyday life (Charon 2001; Silistraru 2017).

On the other hand, from the humanities, and especially literary studies, physicians can learn how to perform the narrative aspects of their practice with new effectiveness.

Despite being a method, the NBM can give doctors the skills, methods, and texts to learn how to imbue the facts and objects of health and illness with their consequences and meanings for individual patients and physicians.

## Methods

52 destructurated interviews from clinicians involved in COVID activities have been collected in the “Ospedale dei Castelli” hospital in Rome, Italy, from from April 2020 to December 2020. Interview answers were broken down in elementary themes, and subsequently analysed in order to obtain a conceptual framework, using the technique of Narrative Analysis.

On the basis of the raw data collected through the interviews, a triple classification was carried out, exploiting the principles of Content Analysis and Thematic Analysis. Content Analysis (Krippendorff 2012) is used to identify factors affecting the way in which interviewees organize their emotional and first-hand experience in their answers, and to categorize these factors into attributes (Chiu 2005). This type of analysis may help researchers with large amounts of text data, as content analysis is useful for determining how words and word patterns are used in context. The thematics employed in classification, and the connections between them, provided the basis for a fresh description. This analysis made it possible to have a first classification of the answers, based on the most common feelings developed by health personnel employed in Covid wards during the syndemia.

Collected data were used to build an interpretative model of Covid phenomenology in relation to healthcare professionals and patients, articulated by the 5 driving forces recognized by the ancient world (Love, Anger, Envy, Fear and Thirst for Knowledge) as described by Ovid. A structured approach was initially used, by providing the main framework with interview questions. For each question, similar answers given by different interviewees were grouped and were assigned an emergent code: Positive driving forces, or Negative driving forces.

Since all textual data within in an interview revolves around some major themes, and since all interviews are somehow related, representing different dimensions of the same phenomenon, albeit from different perspectives, Thematic analysis was employed to investigate which aspects participants were more prone to talk about, either by frequently referring to or by having a depth emotional involvement, and to investigate the ways those aspects could be connected each other. Thematic analysis as a tool is already well-established in the field of narrative research, and has been proved to be a potentially reliable source of evidence in this field (Nowell et al. 2017).

Narrative analysis brought to the identification of components within the four major narrative scenarios of Narrative Based Medicine (Physician and Patients, Physician and Self, Physician and Colleagues, Physician and Society), based on the data collected via in-person interviews. For each four narrative scenarios the associations with positive and negative driving forces were investigated in the second phase.

## Results

The model attributed a specific meaning to each of the 5 forces, contributing to the phenomenon interpretation from an exclusive sociological and sanitary point of view, within the four major personal relations: Physician and Patients, Physician and Self, Physician and Colleagues, Physician and Society.

The conceptual understanding of the unpredictable forces that reign over us seemed strangely apt when trying to define the Coronavirus “Tsunami” as seen from a front-line medic’s point of view.

Love is so evident in every image transmitted by the mass media from all over the world: the passion of doctors, nurses and support staff shielded from head to toe in protective gear, all part of the same global army battling an invisible and perilous foe.

Anger, which has fueled aggressive reprisals during the lockdown, despite our momentary transformation into angels and heroes, and which may be a presage of future retaliation for cures that we could not guarantee or for the use in good faith of medications for off-label indications.

Envy generated by isolation which, by negating the fundamental human right of personal liberty, far from engendering solidarity rather awakened primordial behaviors; the impulse to escape, aggression, distancing oneself emotionally from the horror: as the old saying “mors tua, vita mea”. Indeed, the survival instinct can inspire senseless acts of selfishness, crushing the bonds of family and friendship.

Fear is the sentiment that burns in the eyes of those who surround us and in particular on the faces of sick patients who are unsure of what fate awaits them, fear of death, fear of suffocation, fear of the unknown (Faust and del Rio 2020).

Thirst for Knowledge may represent the most contemporary of the five, and from the beginning of the pandemic has produced an immense quantity of scientific information acquired and vetted in an entirely innovative way, in order to share and divulge best practice protocols and to arm us with the facts to combat fake news.

**Fig. 1:**
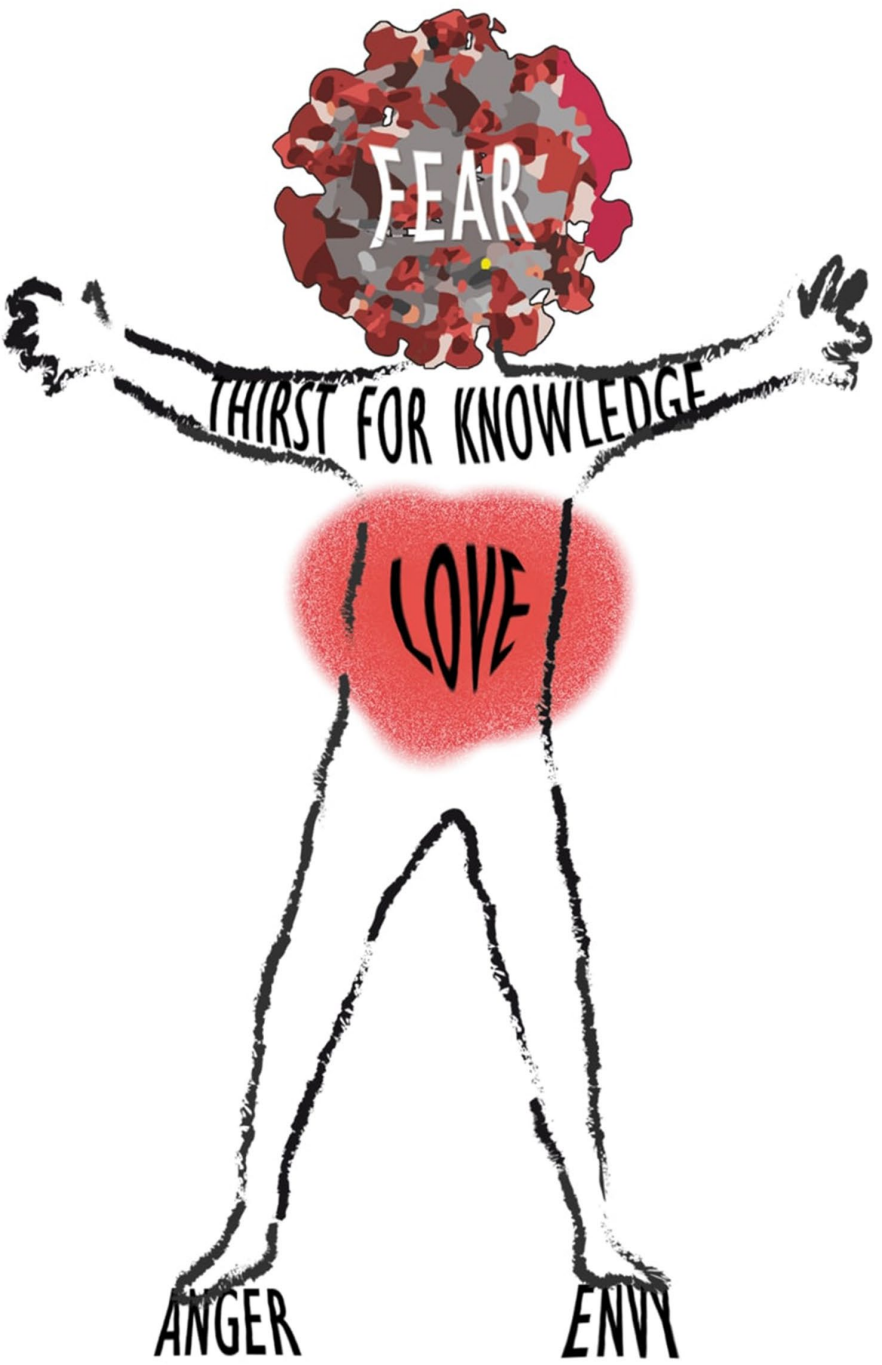
“Individual in times of Covid”. Fear envelopes every thought as our vision becomes binary: “Covid or non-Covid” (Shakespeare, Hamlet. A. Lombardo Ed. Feltrinelli 2013). Thirst for Knowledge is represented as an open embrace, kindled by underlying Love, the great beating heart of health workers’ tireless efforts to save lives no matter what the challenge, finally revealed to all. Anger and Envy are distant from the head and heart, where negative feelings distorted and amplified by the sense of danger risk forming the base of an individual’s actions

These aspects are personified in Fig. 1.

For each four narrative scenarios an association with Ovid’s positive and negative driving forces has been investigated. It is possible to associate at least one positive driving force and one negative driving force to each scenario, as shown in Table 1.

**Table 1:** Correlation between the four major narrative scenarios of Narrative Based Medicine and Ovid's five forces

	Positive driving force	Negative driving force
Physician and Self	Thirst for Knowledge	Fear
Physician and Colleagues	Thirst for Knowledge	Envy
Physician and Patients	Love	Anger
Physician and Society	Love	Fear, Anger

## Physician and self

What has Coronavirus meant for me? A true metamorphosis which has completely changed my clinical perspective. As an internist, specialized in healthcare management, and usually quite at ease working in a modern and dynamic Medical Department, I suddenly found myself grappling with the label “COVID ward”, having to transform an Internal Medicine department into a fully functional Isolation Unit (World Health Organization. Clinical management of severe acute respiratory infection when novel coronavirus (2019-nCoV) infection is suspected 2019).

Fear is the main feeling, and Thirst for Knowledge represents the only way to fight against it, by trying and providing answers to all questions that pandemic posed to healthcare professionals.

## Physician and colleagues

New strengths were discovered, including the courage of a team eager to face into the challenge this new disease presented, both mentally and physically, and boosted by the fearless contribution of new, young colleagues. They never lost sight of the holistic approach to patient care while rapidly acquiring new skills essential in combatting this perilous foe. Indeed, this illness never gave us a chance to draw breath for a moment ourselves, as the presentation and treatment hypotheses changed at dizzying speed, constantly forcing us to modify and adapt established procedures and our behavior as the pandemic evolved (WHO: Clinical management of severe acute respiratory infections when novel coronavirus is suspected: What to do and what not to do. 2020). And never forgetting the dark shadow of “physician burnout” (Queen and Harding 2020; Hartzband and Groopman 2013), ever ready to ambush, picking off one of my most valued colleagues. As such although we escaped physical infection, this virus has penetrated and marked us both personally and professionally, as we struggled to keep up with its capricious and mercurial nature.

This relation is based and positively enhanced on sharing information and developing synergies and collaboration among professionals; it is negatively affected by envy that may contaminates relationships between colleagues.

## Physician and patients

A “Covid” Story. Mrs. C’s story is typical of the “Metamorphosis” that this clinical and social entity has come to represent (Nonis and Di Virgilio 2020). This patient, a 75-year-old woman with both aortic and mitral prosthetic valves, had been suffering from recurrent episodes of fever of undetermined origin. One of these episodes occurred during the Covid pandemic leading to obligatory admission to the Covid ward, despite repeated negativity of molecular tests. A seemingly straightforward decision given the “unequivocal” radiological signs of Covid pneumonia (Kooraki et al. 2020). The relatively “banal” differential diagnosis of endocarditis (Hoen and Duval 2013) was rapidly excluded by a FAST scan in Emergency Room (ER). The admission lasted over two weeks, during which her respiratory problems seemed prevalently linked to acute heart failure. Serological testing confirmed a recent contact with the virus. Having spoken to the patient and her family my inner internist (no pun intended) became increasingly more convinced that the underlying problem was endocarditis; here the rigid barriers erected to protect instead impeded; a transesophageal echocardiogram was out of the question. Her clinical picture improved enough to allow discharge home, however she soon presented again to ER with violent fever. On readmission the echocardiogram and blood cultures finally confirmed endocarditis of the aortic valve due to Escherichia Coli. However, the Covid label cannot be shed lightly, and only a joint consultation between internist and infectious disease specialist finally allowed the patient, whose condition had in the meantime deteriorated and now required inotropic support, to safely be transferred to a sub-intensive cardiology unit.

In the complex relationship between physician and patient Love is the dominating feeling. Anger onsets on burn-out, when the weight of responsibilities and requested commitment overcome the individual’s resources and coping capacity.

## Physician and society

The Covid era has been defined by some important precedents; including the lack of preparedness for an emergency of this nature (Investing in and building longer-term health emergency preparedness during the COVID-19 pandemic interim guidance for WHO Member States 2020), together with a void in terms of international coordination and cooperation between States, exasperated by the progressive defunding of healthcare systems. In addition, media reports generating terror and contributing to confusion and disinformation risk weakening the resolve of populations, who have accepted significant limitations to their personal freedom in the name of preserving life.

Physician is classically represented as an expression of love towards others, however due to the pandemic he feels Fear for the potential consequences of his own work, and Anger—coming from fear—when he is forced to defend himself from both political power, accusing him of not being able to find adequate and secure answers in a short time, and from patients and caregivers who base their relation on threat and aggression rather than trust and gratitude, considering him responsible of any unsatisfactory outcome.

## Discussion

In a pre-Covid era the reported “case report” would have been simple, but due to the metamorphosis of a health system which sees only Covid patients while the others “can wait” (Rosenbaum 2020), the management of this patient suddenly became unfathomly complex (Driggin et al. 2020). However, this case in reality was another in a series of learning experiences, calling into play not only our clinical acumen but our ability to handle frustrating and unfamiliar obstacles holistically, methodically (using and interpreting protocols without being overly behoven to them), and by managing to maintain the relationship between the physician and the patient and her family, despite distancing and partitions, in order to reach the diagnosis together.

Many healthcare workers, as I, having come face-to-face with the magnitude of this emergency, are able in some way to contextualize the social implications of this experience, which paradoxically has some positive aspects, having discovered newfound courage, resourcefulness, and hope. On the other hand, those who feel they have been solely subjected to this crisis, isolated from the reality of the situation, tend to scotomize, seeking to recuperate months of normal life that Covid has in some way denied them.

So how can we defeat the Covid crisis? By combatting against the terror of the heart, by finding the passion and courage to face into this difficult situation and above all by dealing honestly with our fear of disease and death. This allows the inner growth that rekindles our ability to fight on, stronger and more aware.

The Covid emergency has found us unprepared not only on an organizational level but also emotionally, and the fear of even contemplating the possibility of our own mortality has revealed our fragility and left us open to manipulation by instant “saviours” offering easy solutions and soft platitudes. However personal courage is within reach of us all, not only those much-applauded front-line workers, and it is with that courage that we face our own fragility in defense of our liberty and freedom of thought.

## Conclusions

Proposed interpretative model comes from the awareness that Literature can suggest ways of interpreting the world surrounding us, and relations are the basis of any event that involves humans.

Confrontation with the 5 forces recognized by Ovid in his Metamorphosis suggest that the main relation of a physician is within itself: only by self-overcoming the fears of illness and death it shall be able to be helpful and useful towards others.

Relationship with patients is a listening relation, whose objective is to set up a personal therapeutic path necessary to obtain diagnosis and healing.

Healthcare workers job is impossible without some sort of team spirit that strengthens, encourages and raise each member's resilience and betters performances quality.

Lastly, mass media, coupled with lack of international coordination, showed us that relation between physicians and society is frail and compromised.

Cultural authority has given way to a disseminated information that can be retrieved with speed and ease and is understandable to all, but often contradictory.

Patient's history, however, leaves us with a message of hope: if we listen what the patient says, even in harsh and confused conditions, we will be able to build together a healing path narrating a history as unique as every human being.

“If you wish to see the rainbow, you must learn to love the rain” Paulo Coelho.
